# Regulation of Cancer Stem Cell Metabolism by Secreted Frizzled-Related Protein 4 (sFRP4)

**DOI:** 10.3390/cancers10020040

**Published:** 2018-01-31

**Authors:** Abhijeet Deshmukh, Frank Arfuso, Philip Newsholme, Arun Dharmarajan

**Affiliations:** School of Pharmacy and Biomedical Sciences, Curtin Health Innovation Research Institute, Curtin University, Perth, WA 6102, Australia; abhijeet.deshmukh@student.curtin.edu.au (A.D.); frank.arfuso@curtin.edu.au (F.A.); Philip.Newsholme@curtin.edu.au (P.N.)

**Keywords:** cancer stem cells, metabolism, secreted fizzled-related protein, glycolysis, glucose, apoptosis

## Abstract

Tumours contain a small number of treatment-resistant cancer stem cells (CSCs), and it is through these that tumour regrowth originates at secondary sites, thus rendering CSCs an attractive target for treatment. Cancer cells adapt cellular metabolism for aggressive proliferation. Tumour cells use less efficient glycolysis for the production of ATP and increasing tumour mass, instead of oxidative phosphorylation (OXPHOS). CSCs show distinct metabolic shift and, depending on the cancer type, can be highly glycolytic or OXPHOS dependent. Since Wnt signalling promotes glycolysis and tumour growth, we investigated the effect of the Wnt antagonist secreted frizzled-related protein 4 (sFRP4) on CSC metabolism. We demonstrate that sFRP4 has a prominent role in basal glucose uptake in CSCs derived from breast and prostate tumour cell lines. We show that sFRP4 treatment on CSCs isolated with variable glucose content induces metabolic reprogramming by relocating metabolic flux to glycolysis or OXPHOS. Altogether, sFRP4 treatment compromises cell proliferation and critically affects cell survival mechanisms such as viability, glucose transporters, pyruvate conversion, mammalian target of rapamycin, and induces CSC apoptosis under conditions of variable glucose content. Our findings provide the feasibility of using sFRP4 to inhibit CSC survival in order to induce metabolic reprogramming in vivo.

## 1. Introduction

Accumulating evidence suggest that tumours of various tissue origins, including breast, prostate, and ovary contain a small sub-population of cells with stemness capacity, often referred to as cancer stem cells (CSCs) or tumour initiating cells [1,2,3,4]. In addition to the CSCs’ self-renewal and migratory capacity, they also possess the ability to efflux toxic compounds and chemotherapeutic agents due to their high expression of ATP-dependent efflux pump ABCG2, high DNA repair system, and activation of survival cascades [5,6,7]. 

Current research has established certain key components and signalling pathways that affect the stemness and differentiation of CSCs [8,9,10,11], although the effect of nutrients and metabolites on CSCs remains elusive. A recent study suggests that CSCs have particular metabolic properties enabling their identification from the bulk tumour cells based upon their biochemical profile [12]. Another study demonstrated that brain CSCs exhibit low mitochondrial respiratory activity and prefer a hypoxic environment to maintain their stemness [11]. Glioma stem cells (GSCs) were glycolysis driven and were intrinsically sensitive to the use of a glycolytic inhibitor [13]. However, cancer cells prefer glycolysis for their ATP production, and CSCs appear to have higher glycolytic activity. The Warburg hypothesis is consistent with the CSCs’ dependency on glycolysis and switching on oxidative phosphorylation to facilitate cytosolic glycolysis [14,15]. Based on these observations that glucose is an essential nutrient for CSCs, we reasoned that glucose might have a significant effect on the CSC subpopulation in bulk tumour cells. Furthermore, this enabled us to evaluate CSC survival under conditions of variable glucose content, and we also investigated the role of Wnt antagonism in regulating CSC survival under these conditions. 

Wnt signalling plays an important role in tissue development and maintenance of normal tissues, though aberrant Wnt signalling activation is implicated in many cancers [16]. Wnt signalling in the “ON” state leads to active β-catenin accumulation in the nucleus, and its interaction with LEF/TCF (lymphoid enhancer factor/T-cell factor) transcription factors leads to activation of Wnt target genes that are important for cancer cell survival. Aberrant Wnt signalling has been implicated in tumorigenesis, as its downstream targets are involved in cell survival, differentiation, and proliferation; therefore, inhibition of the Wnt pathway is a potential strategy for halting tumour progression. Secreted frizzled-related protein 4 (sFRP4) is a Wnt antagonist inhibiting canonical Wnt signalling by binding to Wnt ligands and frizzled receptors [17]. Our previous studies have identified sFRP4’s ability to inhibit multiple functional outputs of oncogenic Wnt signalling in CSCs, including a decrease in viability and epithelial-mesenchymal transition (EMT) induction, inhibiting angiogenesis, inducing apoptosis, and modulating cell survival [8,18]. Here, we propose a novel function of sFRP4 in the regulation of CSC metabolism. 

In this study, we used cell lines from breast (MDA231 and MCF7) and prostate (PC3 and LnCap) tumours to isolate CSCs as an in vitro model of endocrine-related tumours. We investigated the effect of sFRP4 on CSCs isolated in culture medium with no, low, and high glucose content. The addition of glucose to the culture medium induced a significant increase in CSC viability, which was decreased post sFRP4 treatment under the same conditions. We also demonstrated that the CSC metabolic profile changes with increasing glucose content in culture medium, and Wnt signalling plays a key role in mediating glucose induced CSC survival. Finally, we investigated the potential therapeutic effect of sFRP4 on CSC viability, glucose uptake, glutamine uptake, glutamate secretion, NAD+/NADH ratio, and metabolically relevant proteins, and showed that sFRP4 compromised CSC viability and impaired CSC survival by initiating apoptosis regardless of glucose content.

## 2. Results

### 2.1. The Sphere Forming Capacity of CSCs Is Reduced by sFRP4 Irrespective of Glucose Content

**Figure 1:**
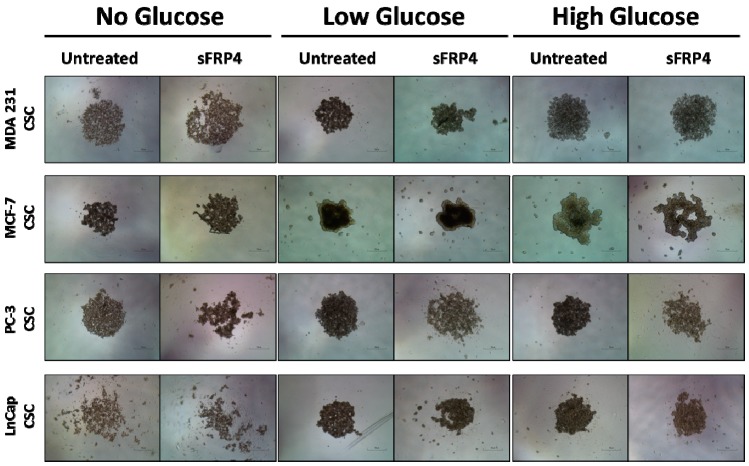
Effect of Secreted Frizzled Related Protein-4 on Cancer Stem Cells morphology: CSCs were isolated from breast and prostate tumour cell lines with increasing glucose concentrations and treated with sFRP4 (250 pg). The sFRP4 treatment results in disruption of the CSC sphere. (Scale bar: 100 μm). Images are representative of all the experiments.

To investigate the potential phenotypic changes that glucose dependency might induce in CSCs, we evaluated CSC morphology in different culture media containing varied glucose levels. CSCs were treated with sFRP4 (250 pg) for 24 h. The untreated spheroids remained intact, whereas the sFPR4 treated cells showed disruption of spheroids (Figure 1) in No Glucose/Low Glucose/High Glucose culture medium. However, sFRP4 segregated the spheroids in MCF-7, PC-3, and LnCap CSCs (Figure 1). Without glucose, CSCs spheroids are more stressed and susceptible to sFRP4’s segregation capacity. CSCs isolated in low glucose culture medium showed a similar effect as no glucose, indicating that glucose is critical for CSC survival. However, sFRP4 segregated the spheroids in all glucose conditions, indicating that sFRP4’s effects are not influenced by glucose conditions.

### 2.2. CSC Viability Is Reduced by sFRP4 in Low and High Glucose Conditions

**Figure 2:**
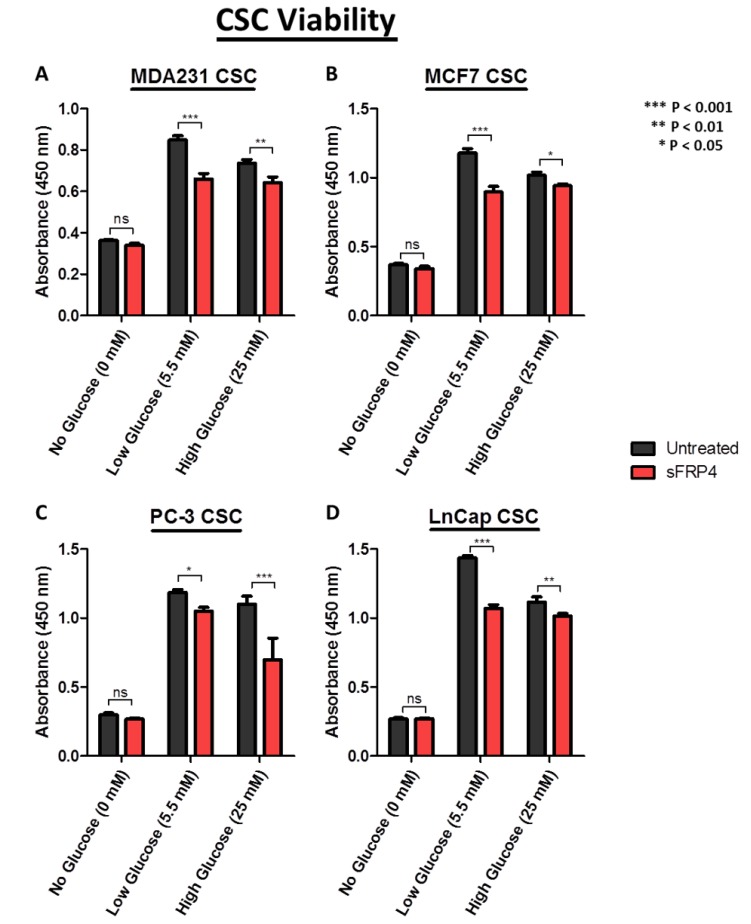
Effect of sFRP4 on CSC viability: Viability assay was performed using Cell Counting Kit-8 after treatment of CSCs derived from (**A**) MDA231; (**B**) MCF7; (**C**) PC-3; and (**D**) LnCap cell lines treated with sFRP4 for 24 h. Statistical analysis was performed using ANOVA for analysis variance with Bonferroni test for comparison showing significance as *** *p* < 0.001; ** *p* < 0.01; * *p* < 0.05. Data are mean ± standard error of mean from three independent experiments.

Using a Cell Counting Kit-8 assay, it was observed that the viability of CSCs increased with increasing glucose content in the culture medium (Figure 2).

This indicates the requirement of glucose for CSC metabolism and survival. However treatment with sFRP4 significantly inhibited the viability of CSCs in low and high glucose conditions compared to untreated CSCs. In low glucose conditions, sFRP4 critically affected the viability in MDA231 (Figure 2A), MCF-7 (Figure 2B), PC3, and LnCap CSCs (Figure 2D), whereas a minimal effect was observed in no-glucose conditions for all CSCs.

### 2.3. Correlation Coefficient of sFRP4 with Metabolic Gene-Set

**Figure 3:**
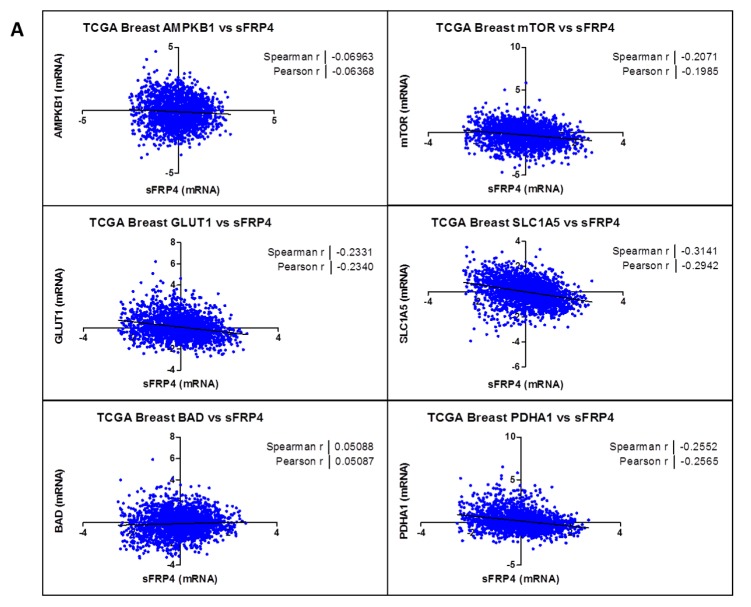

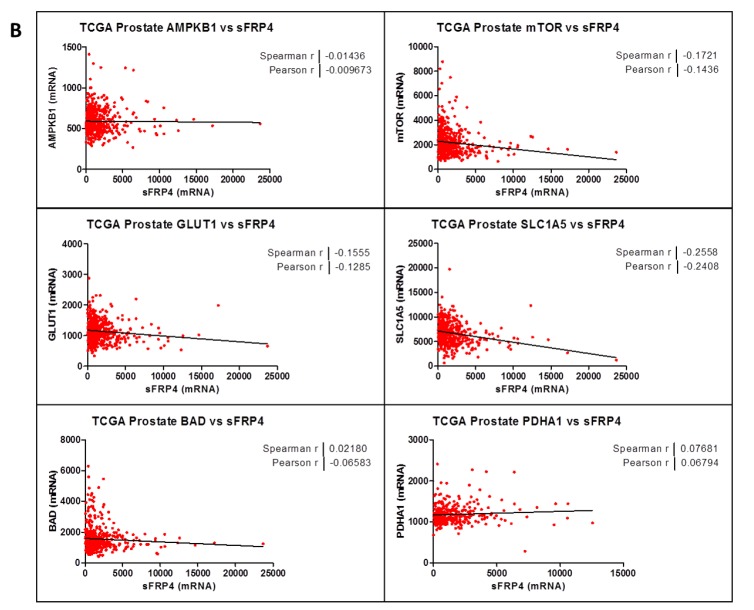
Correlation coefficient of sFRP4 with metabolic gene-set: Spearman and Pearson correlation coefficient between the mRNA expression values from TCGA patient tumour data. (**A**) Breast Invasive Carcinoma (*n* = 825) and (**B**) Prostate Adenocarcinoma (*n* = 333).

Our initial goal was to generate a gene-expression signature representing sFRP4 activation and to use that signature to identify metabolic targets in breast and prostate cancer through The Cancer Genome Atlas. The set of genes was further narrowed by identifying the subset of genes with established or putative roles in cancer metabolism. Because some of the genes were identified as cancer amplified genes, we further analyzed the gene set to identify putative metabolism driver genes. This was done by calculating the Spearman and Pearson correlation coefficient between the mRNA expression values from The Cancer Genome Atlas (TCGA) patient tumour data. Correlation coefficients were calculated for both breast (Figure 3A) and prostate (Figure 3B) cancer, and average correlations were calculated for each gene. The gene set comprised metabolic genes such as *AMPKB1* (AMP Kinase), *mTOR* (mammalian target of rapamycin), *GLUT1* (glucose transporter), *SLC1A5* (glutamine transporter), *BAD* (Bcl-2 associated death promotor), and *PDHA1* (pyruvate dehydrogenase). The analysis revealed a negative correlation coefficient between *sFRP4* and the gene set, indicating an inhibitory effect of *sFRP4* on all those genes. The only gene to have a positive correlation coefficient was *BAD*, indicating the pro-apoptotic capacity of sFRP4 in cancer metabolism in both the tumours examined. Although *AMPK* showed minimal correlation, it plays an important role in regulating the PI3K/AKT/mTOR signalling cascade, and we decided to include this gene set for our protein modification study. 

### 2.4. The Effect of Glucose and sFRP4 on Glucose-Uptake in CSCs

**Figure 4:**
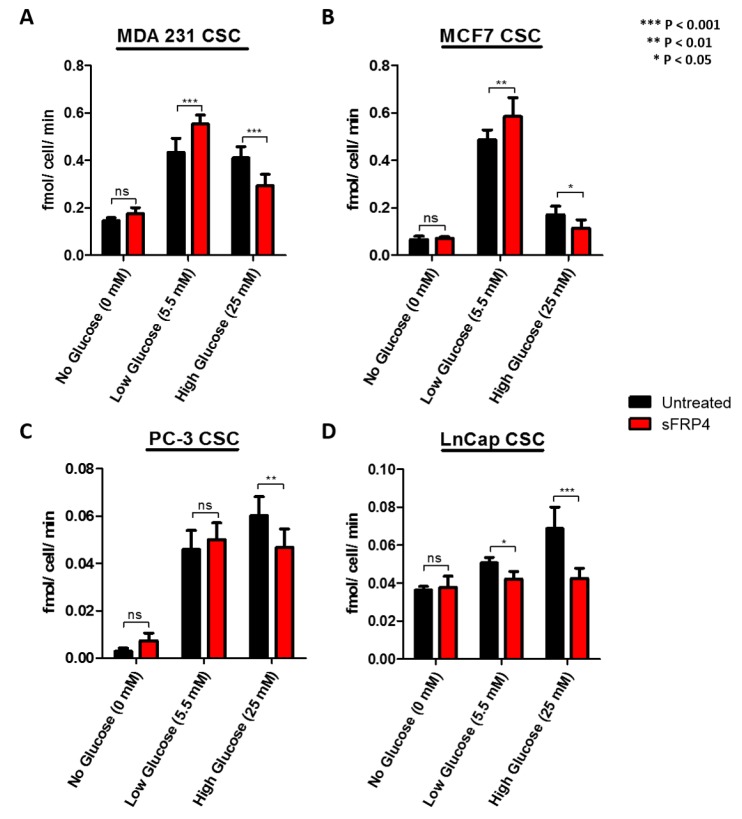
The effect of sFRP4 on glucose-uptake in CSCs: CSCs were isolated in various glucose concentrations and treated with sFRP4 (250 pg) for 24 h. Cellular uptake was detected with Glucose Uptake-glo assay. (**A**) MDA231 CSCs; (**B**) MCF7 CSCs; (**C**) PC-3 CSCs; and (**D**) LnCap CSCs. Statistical analysis was performed using ANOVA for analysis variance with Bonferroni test for comparison showing significance as *** *p* < 0.001; ** *p* < 0.01; * *p* < 0.05. Data are mean ± standard error of mean from three independent experiments.

To better understand the effect of glucose on glycolytic metabolism, the CSCs were isolated in various glucose concentrations, and cellular uptake was detected with a glucose uptake-glo assay (Figure 4). Glucose induced an increase in glucose uptake by 2-fold in MDA231 CSCs, 5 folds in MCF7 CSCs and PC3 CSCs low glucose groups, whereas in the high glucose groups there was a 2 folds increase for MDA231 CSCs and LnCap CSCs, and 6-fold in PC3 CSCs, compared with the no glucose groups (Figure 4A–D). Breast CSCs had higher glucose uptake in the low glucose groups (Figure 4A,B); whereas prostate CSCs had higher glucose uptake in the high glucose groups (Figure 4C,D). We then monitored the effect of sFRP4 on glucose uptake. The levels of glucose uptake in low glucose groups significantly increased in all CSCs, whereas they decreased significantly in high glucose groups, suggesting that sFRP4 has a direct effect on glycolytic flux and in increasing the glycolytic activity. 

### 2.5. Changes in Extracellular Metabolites with sFRP4 Treatment of CSCs

**Figure 5:**
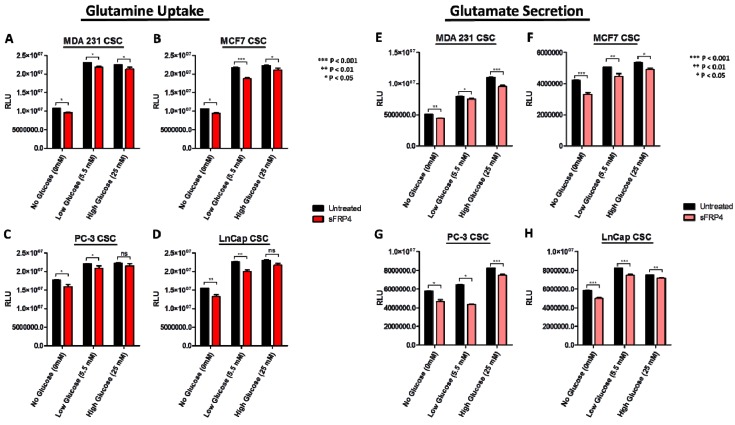
Extracellular metabolites with sFRP4 treatment of CSCs: Changes in glutamine uptake and glutamate secretion in CSCs grown in culture medium with increasing glucose concentrations were measured. (**A**,**E**) MDA231 CSCs; (**B**,**F**) MCF7 CSCs; (**C**,**G**) PC-3 CSCs; and (**D**,**H**) LnCap CSCs were treated with sFRP4 for 24 h. Statistical analysis was performed using ANOVA for analysis variance with Bonferroni test for comparison showing significance as *** *p* < 0.001; ** *p* < 0.01; * *p* < 0.05. Data are mean ± standard error of mean from three independent experiments.

To further characterize the metabolic profiles of CSCs, changes in extracellular metabolite levels during CSC growth in culture were measured. The starting concentration of glutamine is consistent in all CSCs medium, and as cells grow they consume these metabolites. During growth, glutamate is secreted and accumulated in the medium. The CSCs consumed glutamine and secreted glutamate with time and cell density dependence. Glutamine consumption was more robust in CSCs isolated in all glucose groups (Figure 5A–D). The glutamine uptake increased with increasing glucose content in the medium. In contrast, glutamate secretion (Figure 5E–H) was also observed in all CSCs, indicating glutaminolysis activity. The relative luminescence units (RLU) increased in glutamine uptake with increase in glucose content, indicating the co-activity of glycolysis and glutaminolysis. However, glutamate secretion was significantly different in all the CSCs. Upon addition of sFRP4, we observed a decrease in glutamine uptake and glutamate secretion in all CSCs. However, PC3 CSCs (Figure 5C, G) exhibited a marked effect following sFRP4 treatment, as we observed a higher inhibition of extracellular metabolite secretion. 

### 2.6. Changes in Redox Signature with sFRP4 Treatment of CSCs

**Figure 6:**
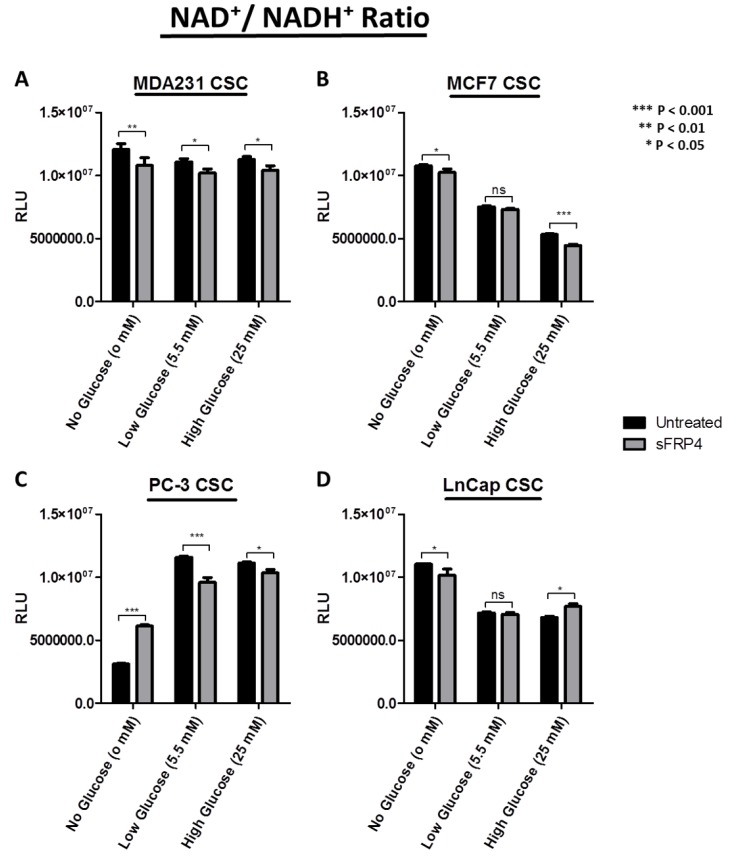
Redox signature with sFRP4 treatment of CSCs: Comparison of NAD+/NADH ratio in CSCs with no, low, and high glucose content. (**A**) MDA231 CSCs; (**B**) MCF7 CSCs; (**C**) PC-3 CSCs; and (**D**) LnCap CSCs were treated with sFRP4 for 24 h. Statistical analysis was performed using ANOVA for analysis variance with Bonferroni test for comparison showing significance as *** *p* < 0.001; ** *p* < 0.01; * *p* < 0.05. Data are mean ± standard error of mean from three independent experiments.

The nicotinamide adenine dinucleotide redox couple (NAD+/NADH) is a marker of catabolism. Here we aimed at describing the divergent redox profile of the CSC population isolated from breast and prostate tumour cell lines under different glucose concentrations. We also demonstrated the effect that sFRP4 has on the divergent redox signature of these CSCs (Figure 6). The results show the comparison of NAD+/NADH ratio in CSCs with no, low, and high glucose content, and the effect of sFRP4 in these conditions. The NAD+/NADH ratio was considerably higher in MDA231 CSCs, and sFRP4 treated CSCs showed a significant decrease (Figure 6A). The NAD+/NADH ratio in MCF-7 CSCs gradually decreased with an increase in glucose concentration, whereas sFRP4 had a minimal effect (Figure 6B). In PC3 CSCs, the NAD+/NAD ratio had an inverse activity as compared to MCF7 CSCs; here the ratio increased with an increase in glucose concentration, and sFRP4 treatment oscillated CSC catabolism by increasing the ratio in the no-glucose group, whereas it decreased the ratio in low and high-glucose groups (Figure 6C). The NAD+/NADH ratio in LnCap CSCs followed this trend, and decreased the redox activity as the glucose concentration increased; moreover, sFRP4 had no aberrant effect and decreased the ratio in two out of three glucose groups (Figure 6D). The generalised observation was that hormone-independent CSCs such as MDA231 (ER−) and PC3 (AR−) demonstrated a higher NAD+/NADH ratio with increasing glucose concentrations (Figure 6A,C); whereas hormone-dependent CSCs MCF7 (ER+) and LnCap (AR+) demonstrated a decrease in the NAD+/NADH ratio with an increase in glucose concentration (Figure 6B,D). 

### 2.7. The Effect of sFRP4 on CSC Metabolism Target Proteins

**Figure 7:**
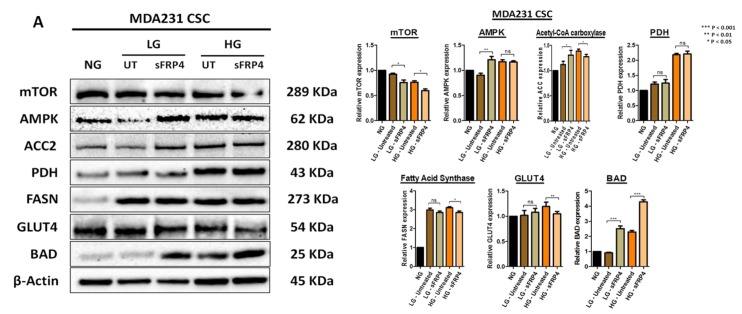

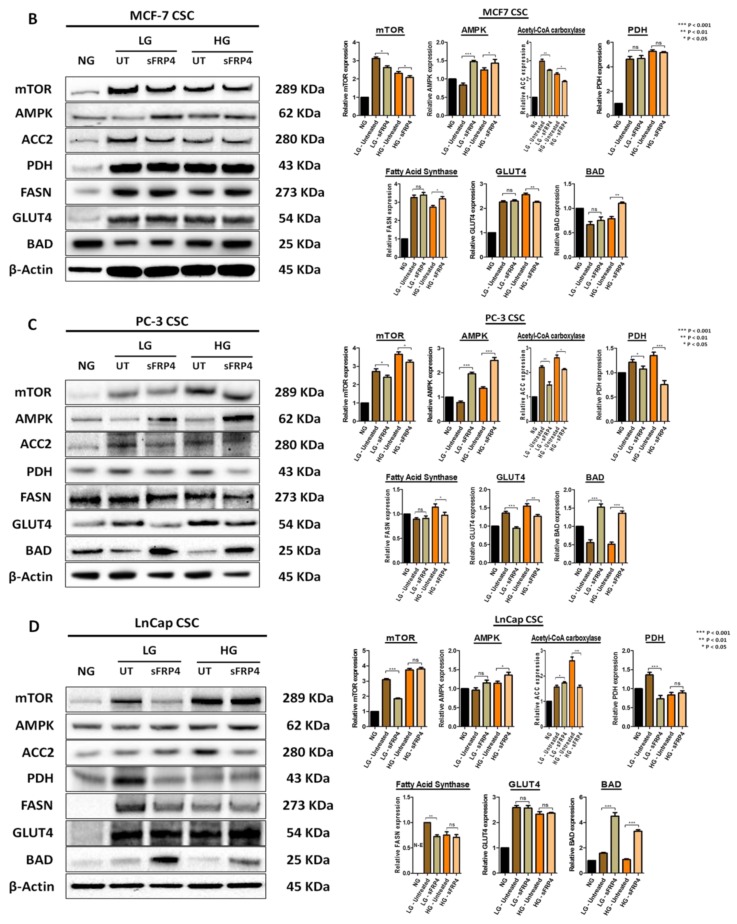
The effect of sFRP4 on CSC metabolism target proteins: Changes in CSC metabolic profile and the post-translational modifications with increasing glucose conditions. (**A**) MDA231 CSCs; (**B**) MCF7 CSCs; (**C**) PC-3 CSCs; and (**D**) LnCap CSCs were treated with sFRP4 for 24 h. Densitometry analysis was performed using ANOVA for analysis variance with Bonferroni test for comparison showing significance as *** *p* < 0.001; ** *p* < 0.01; * *p* < 0.05; *ns*—non-significant. Blots and relative protein expressions are mean ± standard error of mean from three independent experiments.

Following CSC isolation in different glucose concentrations and sFRP4 treatment, we investigated the post-translational modifications in CSCs for a central regulator of cell metabolism (mTOR), AMP-activated protein kinase (AMPK), rate-limiting enzyme (acetyl-CoA carboxylase 2), metabolic oncogene (fatty acid synthase), metabolic gatekeeper (pyruvate dehydrogenase), glucose transporter (GLUT4), and Bcl-2 associated death promotor [19]. MTOR was highly expressed in all the untreated groups but decreased when treated with sFRP4 in low and high glucose groups, except for the LnCap CSC high glucose group (Figure 7D). 

The AMPKα protein levels were observed in all the glucose groups and were significantly elevated with sFRP4 treatment, except the MDA231 CSC high glucose group (Figure 7A). This indicates the AMPKα activity in regulating mTOR, indicating the role of AMPK in the PI3K/AKT/mTOR signalling cascade. The protein levels of acetyl-CoA carboxylase 2 (ACC2), a main isoform in lipogenic and oxidative tissues, decreased in all CSC high glucose groups following sFRP4 treatment, whereas there was an increase post-sFRP4 treatment in the MDA231 CSC low glucose group (Figure 7A). Overexpression of pyruvate dehydrogenase confers higher pyruvate conversion to acetyl-CoA; however, sFRP4 treatment significantly decreased ACC2 protein expression in PC3 CSCs for all glucose groups (Figure 7C) and LnCap CSC low glucose groups (Figure 7D). There was a reduction in fatty acid synthase (FASN) protein expression following treatment with sFRP4 in high-glucose groups; however, there was a minimal effect in all the CSCs in low-glucose groups except LnCap CSCs. The glucose transporter GLUT4 protein expression in all CSCs increased with an increase in glucose concentration; however, GLUT4 decreased with sFRP4 treatment in all CSCs in the high glucose group, and only the PC3 CSCs low-glucose group exhibited a decrease in GLUT4 expression post-sFRP4 treatment (Figure 7C). Expression of Bcl-2 associated death promotor BAD was lower in untreated CSCs but increased significantly with sFRP4 treatment across all glucose groups. The increased BAD protein expression confirms the pro-apoptotic role of sFRP4.

## 3. Discussion

It has been established for decades that cancer cells actively use glycolytic metabolism even in the presence of oxygen, which is known as the Warburg effect [14,15]. The cancer cells are benefited by the high glycolytic rate for ATP production and other metabolites [20]. Due to this prevalent glycolytic elevation in cancer cells and its clinical relevance, such metabolic alterations have been considered as the hallmark of cancers [21]. Recent studies suggest that CSCs may have even more active glycolytic activity compared to the bulk of tumour cells [11,13,22]. Consistently, the current study showed the CSCs to be highly glycolytic, and these observations underscore the importance of glucose as an essential nutrient for CSCs and suggest the possibility that levels of glucose in the tumour microenvironment might significantly affect CSC survival. Therefore, the knowledge of CSC metabolism is of great importance for our understanding of reproductive tumours (i.e., breast and prostate), which are tumours with poor prognosis. To gain further insights into CSC metabolism, we investigated the role of sFRP4 on CSCs isolated in culture medium with various glucose concentrations. Our study demonstrates sFRP4 elicits an anti-proliferative effect and causes spheroid disruption, and decreases glucose uptake, glutamine uptake, glutamate secretion, redox signature, and the signalling cascade responsible for cell survival, and also promotes apoptosis within the CSCs, therefore indicating sFRP4’s potential role in regulating CSC metabolism. 

While the important role of tumour tissue niches in affecting CSCs has gained attention in recent years, the impact of key nutrients in the tumour microenvironment remains largely unknown. The CSC niches preserve the CSCs’ phenotypic plasticity, facilitate metastatic potential, and support high expression of drug efflux transporters, making them highly chemo-resistant [23]. In this study, we used various functional assays to evaluate the effect of glucose and sFRP4 on CSCs, and used Western blot analyses to investigate the underlying mechanisms. Our results exemplify that glucose plays a major role in promoting the CSC phenotype (Figure 1). We also showed that targeting the Wnt signalling pathway by using sFRP4 has the capacity to disrupt the CSC niches in various glucose concentrations (Figure 1). CSCs in a glucose depleted medium were morphologically stressed and more susceptible to sFRP4. Spheroid disruption by sFRP4 decreases the CSCs’ plasticity and cell-cell adhesion, initiating the CSCs’ differentiation towards tumour cells and reducing their self-renewal capacity. The induction of CSCs by glucose appears to be a reversible phenomenon. As shown in Figure 1 and Figure 2, switching CSCs from a no glucose medium to a low glucose or high glucose medium led to an increase in their viability and they became morphologically more robust. Consistently, glucose deprivation caused rapid depletion in CSC viability, which re-appeared when glucose was replenished. 

In previous studies, our group has shown that sFRP4 has an anti-proliferative capacity in CSCs derived from breast, prostate, ovary, glioblastoma multiforme, and head and neck tumours [8,24,25]. In this study we demonstrated that sFRP4 decreased the viability of CSCs in increasing glucose concentrations when compared to CSCs in a glucose depleted medium (Figure 2), indicating sFRP4s’ anti-proliferative capacity is independent of exogenous key nutrients in the microenvironment. 

Most cancer cells rely more on glycolysis rather than on oxidative phosphorylation for glucose metabolism [14]. The active utilization of glucose by tumour cells constitutes the basis of 2-[^18^F]fluoro-2-deoxy-d-glucose positron emission tomography (^18^FDG-PET) imaging for cancer diagnosis, and positive FDG-PET signals post-treatment predict poor prognosis [26,27,28,29]. CSCs are chemo-resistant cells, and it is possible that with conventional chemotherapeutic treatment the residual lesions within the tumour would enrich for CSCs with elevated glycolytic activity. In a previous study, CSCs were isolated from human non-small cell lung carcinoma (NSCLC) and colon cancer cell lines [30]. Using flow cytometry, they compared the glucose metabolic activity between CSCs and non-CSCs; glucose uptake was significantly increased in CSCs compared to non-CSCs, indicating that CSCs were more glycolytic than their normal counterparts. To better understand the effect of glucose on glycolytic metabolism, we isolated CSCs in glucose deprived medium and increasing concentrations of glucose. We observed high glycolytic activity within CSCs isolated in low glucose (5.5 mM) medium, and comparatively less in high glucose (25 mM) medium. Meanwhile, we also investigated the effect of sFRP4 on glucose uptake, where we observed varying effects. We postulate that sFRP4 had stressed the CSCs in the glucose deprived medium, driving them to initiate higher glycolytic activity; whereas CSCs in high glucose medium showed an inhibitory effect on glucose uptake, suggesting that sFRP4 has a direct effect on glycolytic flux. Furthermore, the glucose uptake was variable in all CSCs, and no trend was observed. Other studies have also found that there is no differential response to epidermal growth factor [31] and basic fibroblast growth factor (FGF) in the media used to isolate CSCs, ruling out any interference of these growth factors on CSC glucose uptake [32]. Although the presence of glucose in CSC culture medium has been shown to significantly increase the viability of CSCs in the overall isolation process, glucose uptake is an essential process and a key nutrient for CSCs [30]. A recent proteomic and targeted metabolomics analysis between breast CSCs and their counterpart revealed a metabolic pathway associated with the stem-like conditions, indicating that breast CSCs shift from mitochondrial OXPHOS towards fermentative glycolysis [33]. 

Although cancer cells exhibit high rates of glycolysis, their mitochondrial OXPHOS remains intact and becomes progressively more dependent on glutamine metabolism [34]. In cancer cells, the rate of glutamine conversion to lactate is higher compared to normal cells, which represents an alternative metabolic pathway to glucose consumption in a glucose depleted microenvironment [35]. Glutaminase converts glutamine to glutamate through glutaminolysis. Glutaminase and glutamine levels in the cell culture medium correlate with the cancer cell proliferation, whereas glutamate levels are associated with tumour aggressiveness [36]. Moreover, glutamine’s function to promote cell growth is widely dependent on the epigenetic background of the tumour [37,38]. Glutamine depletion induces apoptosis in melanoma and prostate cancer cells, and using acivicin to inhibit glutaminolysis has been very effective in animal models of cancer [39,40,41]. In addition, another study demonstrated the targeting of glutamine uptake as a new therapeutic strategy to treat acute myeloid leukaemia [42]. However, glutamine deprivation of CSCs is less well characterized. Morover, previous evidence has suggested that intra-mitochondrial protein AIF translocates to the nucleus and promotes caspase-independent cell death induced by glutamate toxicity [43]. We demonstrated in our study that sFRP4 significantly decreased the glutamine uptake and glutamate secretion in all CSC glucose groups, indicating the key role of sFRP4 in glucose/glutamine metabolism. Another study demonstrated that glutamine promotes cell growth in ovarian cancer cells by activating the mTOR/S6 and MAPK pathways [44]. This prompts us to suggest that targeting glutaminolysis by sFRP4 might prove a valuable step in regulating CSC metabolism. 

The NAD+/NADH ratio is directly impacted by glycolytic and mitochondrial activities that change during metabolic reprogramming. The NAD+/NADH redox state plays a key role in cancer cell stemness [45]. Nicotinamide, the NAD precursor, protects cells from apoptosis and senescence by accelerating cell proliferation and alleviating oxidative stress. Accordingly, in CSCs, increased glucose metabolism reduces the level of reactive oxygen species (ROS) to promote EMT [10,46], whereas the level of NADH is decreased with a decrease in the ratio of reduced glutathione (GSH) to oxidised (GSSH) glutathione [47]. A high NADH level is a property that is conserved between normal and cancerous stem cells [48]. A previous study has also demonstrated that when CSCs are fed with mitochondrial fuel (l-lactate or ketone bodies), CSCs quantitatively produce more NADH in response to the stimulus compared to non-CSCs [49]. In addition, NAD+ depletion, using the NAMPT inhibitor FK866, potently blocked spheroid formation [48]. We demonstrated a significant reduction in the NAD+/NADH ratio in all CSCs post-sFRP4 treatment, suggesting that a higher NAD+ content is important for enhancing the resistance to stress induced by ROS in CSCs; whereas a decreased NAD+/NADH ratio makes CSCs more susceptible to reprogramming their redox state.

There is growing evidence on the role of the mTOR pathway in the maintenance of CSCs. Prostate cancer radio-resistance is associated with EMT and enhanced CSC phenotypes via activation of the PI3K/Akt/mTOR signalling cascade [50]. Activation of the mTOR signalling pathway enhances breast CSC colony formation ability in vitro and tumorigenicity in vivo [51]. Suppression of mTOR decreases ALDH1 activity in colorectal CSCs [52,53]. In glioblastoma CSCs, cross-inhibitory regulation between the MEK/ERK and PI3K/mTOR signalling cascades contributed to self-renewal and tumorigenic capacity [54]. Aberrant activation of the PI3K/Akt/mTOR signalling pathway leads to an increase in chemokine (C-X-C motif) receptor 4 (CXCR4), which corresponds to maintenance of stemness in NSCLC cells [55]. Interestingly, metformin decreased radio-resistance of CSCs in mouse fibrosarcoma cells and human MCF7 breast cancer cells by activating AMP-activated protein kinase and suppressing mTOR expression [56]. We demonstrated that sFRP4 decreases mTOR protein expression and increases AMP kinase (AMPK) expression, which may inhibit the PI3K/Akt/mTOR signalling cascade via phosphorylation of mTOR. It is possible that the anti-tumour activity of sFRP4 in vitro maybe associated with inhibition of the insulin/IGF-1 pathway through AMPK activation. AMPK regulates mTOR activity through activation of the tuberous sclerosis protein 1/2 complex [57,58]. 

In addition to glucose and glutamine, fatty acids are an important energy source incorporated in extracellular media, or can be obtained endogenously by accumulating lipid droplets [59]. Fatty acid synthesis is an anabolic process, which starts with converting acetyl CoA to malonyl CoA by acetyl CoA carboxylase. We found a higher expression of fatty acid synthase (FASN) in breast CSCs for all glucose groups. Notably, a high expression of FASN has been linked to poor prognosis of pancreatic ductal adenocarcinoma patients and depends heavily on induction of EGFR/ERK signalling [60]. Furthermore, FASN promotes EMT in ovarian [61], breast [62], and colorectal [63] cancers. Inhibition of FASN leads cancer cells to apoptosis, mainly by inhibiting DNA replication and the production of anti-apoptotic proteins [64]. We saw a minimal effect of sFRP4 on FASN, although breast CSCs in the high glucose groups responded with a decrease in FASN, and the LnCap CSC low glucose group had a maximal decrease in FASN. We propose that sFRP4’s effect is associated with the glycolytic switch of CSCs occurring in different glucose concentrations. 

Pyruvate dehydrogenase (PDH) is a key enzyme that mediates the entry of pyruvate to mitochondria where it facilitates its conversion to acetyl CoA. PDH activity is regulated by pyruvate dehydrogenase kinase 1 (PDK-1) [65]. In CSCs, PDK-1 via the TCA cycle, phosphorylates pyruvate dehydrogenase and suppresses the pyruvate to acetyl-CoA conversion. Furthermore, suppressing the metabolic flow of pyruvate in mitochondria induces the conversion of pyruvate to lactate in the cytosol [30,66]. We observed a decrease in Acetyl-CoA Carboxylase (ACC2) expression in CSCs after treatment with sFRP4, indicating the inhibitory effect of the predominant isoform in lipogenic and oxidative tissues and the mitochondrial membrane potential. Higher PDH expression was observed in CSCs in the high glucose group, and sFRP4 had an impairing effect on PDH expression in prostate CSCs. PC-3 CSC treatment with sFRP4 decreased PDH expression in both glucose groups. A recent study revealed that chemical inhibition (via soraphen A) of acetyl CoA carboxylase suppresses self-renewal growth of CSCs derived from the MCF7 cell line [67]. However, the conversion of pyruvate to acetyl CoA in CSCs is still unclear, and how glucose concentration might influence the process is still something worth exploring.

Tumorigenesis is associated with enhanced cellular glucose uptake and increased metabolism. The transmembrane glucose transporter (GLUT) proteins mediate glucose uptake in cancer cells, and initiate the glucose utilisation cascade [68]. GLUT4 is aberrantly expressed in many tumours, though no study has been undertaken within CSCs. We demonstrated that GLUT4 protein levels are increased in all CSCs, and there is increased expression with an increase in glucose concentration. Furthermore, we also observed the inhibitory effect of sFRP4 on GLUT4 protein expression in prostate CSCs. 

Most anti-cancer drugs exert their effect through triggering the apoptosis pathway, although CSCs escape apoptosis by altering their expression levels of pro-apoptotic and anti-apoptotic Bcl-2 family members [8]. BAD (Bcl-2 associated death promoter) is a member of the Bcl-2 family that, when dephosphorylated, initiates apoptosis by heterodimerizing with anti-apoptotic proteins Bcl-xl and Bcl-2 (15). In vivo, BAD phosphorylation was detected in CSCs of 83% breast cancer biopsies [69]. The overexpression of BAD is correlated with chemo-resistance. Interestingly, high-grade tumours exhibit higher BAD protein levels than those with low-grade cancer, suggesting a role in tumour progression [70]. We demonstrated a gradual increase in (dephosphorylated) BAD expression in CSCs treated with sFRP4. Moreover, sFRP4 treatment elevated BAD in all glucose groups. The increased expression of dephosphorylated BAD is an indicator for apoptosis, and an increased expression depicts the activation of caspase cleavage [71]. BAD expression was consistently high in all CSCs treated with sFRP4, and the elevated expression of apoptotic proteins within all the CSCs reinforces sFRP4′s role as a pro-apoptotic agent.

## 4. Materials and Methods

### 4.1. Cell Culture

#### Monolayer Cell Culture

Cell culture plates for adherent cells were purchased from Nunc^TM^ (ThermoFisher Scientific, Waltham, MA, USA). The human breast tumour cell lines MDA-MB 231 (ER−) and MCF-7 (ER+), and human prostate tumour cell lines PC-3 (AR−/PSA−) and LnCap (AR+) were purchased from American Type Culture Collection (ATCC, Manassas, VA, USA). The cells were cultured in RPMI-1640 medium (#11875-093, Gibco, ThermoFisher Scientific) supplemented with 10% foetal bovine serum (#SFBS, Bovogen, Victoria, Australia) and 100 U/mL PenStrep (#15070063, Life Technologies, Carlsbad, CA, USA). All cells were maintained at 37 °C in a humid incubator with 5% CO_2_.

### 4.2. Cancer Stem Cell Isolation 

For CSC isolation, culture plates with an ultra-low-attachment surface were purchased from Corning Life Sciences (Corning, NY, USA). CSCs were cultured in serum-free medium (SFM) containing DMEM-No Glucose (Gibco, US #11966025), DMEM-Low Glucose, 5.5 mM (#SH30021.01, HyClone, South Logan, UT, USA), and DMEM-High Glucose, 25 mM (#SH30081.02, HyClone) supplemented with the growth factors bFGF (20 ng/mL) (#cyt-085, ProSpec Bio, Rehovot, Israel), EGF (20 ng/mL, #cyt-217, ProSpec Bio), and 1× B27 (#17504044, Gibco), and 100 U/mL PenStrep (#15070063, Life Technologies). CSC-enriched populations of cells were obtained by plating a single cell suspension of breast and prostate tumour cells at 10,000 cells/cm^2^ in SFM on Low-adherent six-well plates (#3471, Corning). CSCs were isolated in SFM; the spheroids were formed at the 3rd day of plating tumour cells. To analyse the effects of sFRP4, cells were cultured in medium supplemented with sFRP4 (see Section 4.3 for details). 

### 4.3. CSC Treatment

The CSCs were treated in this study with purified sFRP4 (#1827-SF-025, R&D Systems, Minneapolis, MN, USA). CSC sensitization with sFRP4 was performed by adding sFRP4 to the cell culture at 250 pg/mL [8] for 24 h at 37 °C in a 5% CO_2_ incubator.

### 4.4. Viability Assay

A cell counting viability kit (CCK8, #96992, Sigma-Aldrich, St. Louis, MO, USA) was used for the quantitation of viable cells. Monolayer cells were plated with culture medium varying in glucose content at a density of 10,000 cells/cm^2^ in a low-adherent flat-bottomed 96-well plate (Corning #3474) for 3 days in non-adherent SFM conditions. Wells with treatment-free medium were used as a negative control. CSCs were treated with sFRP4 for 24 h, then 10 μL of CCK8 solution was added to each well and incubated at 37 °C in a 5% CO_2_ incubator for 1 h. Plates were read at 450 nm using an EnSpire Multilabel Plate Reader (Perkin-Elmer, Waltham, MA, USA).

### 4.5. Cell Surface Markers

To assist in determining their identity, cell surface markers were examined in CSCs by flow cytometry (BD FACSCANTO II, BD Biosciences, San Jose, CA, USA) using CellQuest data acquisition and analysis software. APC-CD44 (1:100) (#338805, BioLegend, San Diego, CA, USA), PE Cy7-CD24 (1:10) (#311119, BioLegend), and PE-CD133 (1:100) (#372803, BioLegend). Cells incubated with conjugated irrelevant IgGs were used as negative controls. Tumour specific CSC markers used were: breast CSCs (CD44^⁺^/CD24^−/low^) [4,72] and prostate CSCs (CD133^⁺^/CD44^⁺^) [73]. CSCs were characterized by flow cytometry (BD FACSCANTO II), as previously published [8]. These data are not shown since the surface markers were used only to characterize CSCs. 

### 4.6. The Cancer Genome Atlas Dataset

To analyse the relationship between *sFRP4* and *AMPKB1* (AMP Kinase), *mTOR* (mammalian target of rapamycin), *GLUT1* (glucose transporter), *SLC1A5* (glutamine transporter), *BAD* (Bcl-2 associated death promotor), and *PDHA1* (pyruvate dehydrogenase) in breast and prostate cancers, we obtained data from TCGA, Nature 2011 by using www.cbioportal.org [74,75]. On the home page of the website, select ‘download data’, then select for breast “Breast Invasive Carcinoma (TCGA, Nature 2012)”, and for prostate “Prostate Adenocarcinoma (TCGA, Cell 2015)”, click “mRNA expression Z-score (all genes)” from select genomic profiles and enter gene set for e.g.,: “sFRP4 AMPKB1”, select “Transpose data matrix” and click submit. The sFRP4 and *AMPKB1* (encoding AMPK) mRNA Z-scores for 825 cases (Breast) and 333 cases (Prostate) will appear. The same process was followed for all the genes examined. The correlation between these Z-scores of two genes was then analyzed by Spearman correlation and Pearson correlation, and plotted using GraphPad Prism V5.0 (GraphPad software, La Jolla, San Diego, CA, USA). 

### 4.7. Glucose Uptake in CSCs

The bioluminescent glucose uptake assay was applied to CSCs in 96-well low adherent white luminescent plates. Before beginning the assay, the culture medium was removed and the CSCs were washed with 100 μL of phosphate-buffered saline (PBS). To initiate glucose uptake, 50 μL of 2-Deoxy-d-Glucose (2DG) (1 mM) in PBS was added to cells. The uptake reaction was stopped and samples were processed as described in the standard protocol of the Glucose Uptake Glo Assay kit (#J1342, Promega, Madison, WI, USA). Because glucose uptake is time dependent, the optimal assay time was determined by stopping the reaction at the 90 min point. This is the time frame that was chosen for standard glucose uptake conditions in CSCs. The luminescent signal produced by this assay is proportional to the rate of glucose uptake, but the precise rate of glucose uptake can be calculated by taking into account the number of cells (10,000 cells/well), time of uptake (90 min), and the amount of 2-Deoxy-d-Glucose-6-phosphate (2DG6P) produced (μM), as measured using the standard protocol. The rate of glucose uptake was measured as fmol/min/cell [76]. Luminescence was read with 0.3–1 s integration on a luminometer (EnSpire Multilabel Plate Reader, Perkin-Elmer).

### 4.8. Detection of Extracellular Metabolites in CSC Medium

For measuring changes in glutamine and glutamate in the CSC medium, CSCs were isolated from MDA231, MCF-7, PC-3, and LnCap cells and plated in SFM conditions in low adherent 96-well plates at a density of 10,000 cells/cm^2^. CSCs were grown in 100 μL DMEM medium supplemented with variable glucose concentrations, 4 mM glutamine, and growth factors (see Section 4.2, CSC isolation). The cells were incubated in a tissue culture incubator (37 °C, 5% CO_2_), and were treated with sFRP4 (250 pg) on the 3rd day for 24 h. At the indicated time (i.e., 24 h treatment), 2 μL of culture medium was removed and transferred to a separate 96 well-plate containing 98 μL PBS/well. For metabolite analysis, 4.5 μL of thawed sample was transferred to respective 96 well white luminescent plates for glutamine and glutamate detection. Samples were then assayed as described in Glutamine/Glutamate-Glo Assay (#J8021, Promega) standard protocols [77]. Luminescence was read on a luminometer (EnSpire Multilabel Plate Reader, Perkin-Elmer).

### 4.9. Detection of Qualitative NAD+/NADH in CSCs

The NAD+/NADH ratio was quantified by a luciferase assay provided in the NAD+/NADH Glo Assay kit (#G9071, Promega). CSCs were isolated from MDA231, MCF-7, PC-3, and LnCap cells plated in SFM conditions in low adherent 96-well plates at a density of 10,000 cells/cm^2^. The cells were incubated in a tissue culture incubator (37 °C, 5% CO_2_), and were treated with sFRP4 (250 pg) on the 3rd day for 24 h. Briefly, after appropriate treatment over the desired time, the medium was removed and cells were supplemented with 50 μL of PBS and 50 μL of 0.2 N NaOH solution with 1% DTAB to obtain a cell lysate. To measure NAD+, a 50 μL aliquot of cell lysate was treated with 0.4 N HCL and heat quenched at 60 °C for 15 min. The solution was neutralized with Trizma buffer. NADH samples were heat quenched following the addition of NaOH with 1% DTAB and the solution was neutralized with HCL-Trizma. An equal volume of NAD/NADH-Glo Detection Reagent was added to each well with cell lysate, incubated at room temperature for 60 min, and Luminescence was read on a luminometer (EnSpire Multilabel Plate Reader, Perkin-Elmer).

### 4.10. Western Blotting

CSCs were washed twice with PBS and then lysed in RIPA lysis buffer (#R0278, Sigma) (150 mM NaCl, 1.0% IGEPAL^®^ CA-630, 0.5% sodium deoxycholate, 0.1% SDS, 50 mM Tris pH 8.0, and Proteinase Inhibitor 1×). Post sonication, cell lysates were centrifuged at 14,000 *g* for 10 min at 4 °C, and the supernatants were used for western blotting. The lysates were resolved by sodium dodecyl sulphate-polyacrylamide gel electrophoresis, transferred onto nitrocellulose membranes, and then stained with 0.1% Ponceau S solution (#P3504, Sigma) to ensure equal loading of the samples. After being blocked with 5% non-fat milk for 60 min, the membranes were incubated with primary antibodies mTOR (7C10) (1:1000, #2983, Cell Signaling, Danvers, MA, USA); AMPKα (1:1000, #2532, Cell Signaling); acetyl-CoA carboxylase (C83B10) (1:1000, #3676, Cell Signaling); fatty acid synthase (C20G5) (1:1000, #3180, Cell Signaling); pyruvate dehydrogenase (C54G1) (1:1000, #3205, Cell Signaling); GLUT4 (1:2500, #ab65267, Abcam, Cambridge, UK); BAD (1:500, #sc-8044, SantaCruz, TX, USA); and β-Actin (13E5) (1:1000, #4970, Cell Signaling) overnight at 4 °C, and the bound antibodies were visualized with horseradish peroxidase-conjugated secondary antibodies using the ECL Western Blotting Substrate (GE #RPN2106, Amersham, Pittsburgh, PA, USA) on a Chemi-Doc (Bio-Rad, Hercules, CA, USA) imaging analyser. 

## 5. Conclusions

In summary, sFRP4 plays an important role in breast and prostate CSC metabolism by reducing the CSCs’ proliferative capacity and glucose uptake, modulating their redox signature, and decreasing the CSCs’ survival signalling cascade by targeting the mTOR complex, making them more responsive to therapy. Further in vivo studies may confirm the efficacy of sFRP4 in altering CSC metabolism to prevent tumour relapse and lead to tumour resolution.
